# The Symptoms of Pulmonary Sarcoidosis

**DOI:** 10.3390/jcm12186088

**Published:** 2023-09-20

**Authors:** Marc A. Judson

**Affiliations:** Division of Pulmonary and Critical Care Medicine, Albany Medical College, Albany, NY 12208, USA; judsonm@amc.edu

**Keywords:** sarcoidosis, pulmonary, symptoms, cough, wheeze, dyspnea

## Abstract

The aim of this manuscript is to provide a comprehensive review of the etiology, measurement, and treatment of common pulmonary symptoms associated with sarcoidosis. The assessment of symptoms associated with pulmonary sarcoidosis is an important component of disease management. Some symptoms of pulmonary sarcoidosis are sensitive but nonspecific markers of disease activity, and the absence of such symptoms provides evidence that the disease is quiescent. Although quantifiable objective measurements of pulmonary physiology and chest imaging are important in the assessment of pulmonary sarcoidosis, they correlate poorly with the patient’s quality of life. Because the symptoms of pulmonary sarcoidosis directly relate to how the patient feels, they are reasonable endpoints in terms of clinical research and individual patient care. Recently, the symptoms of pulmonary sarcoidosis are capable of being quantified via patient-reported outcome measures and electronic devices. We conclude that a thorough assessment of the symptoms associated with pulmonary sarcoidosis improves patient care because it is a useful screen for manifestations of the disease, provides insight into the pathophysiology of manifestations of sarcoidosis, and may assist in optimizing treatment.

## 1. Introduction

Sarcoidosis is a multisystem granulomatous disease of unknown cause. The lung is overwhelmingly the most common organ involved, with pulmonary involvement occurring in approximately 90 percent of patients [1,2,3]. The assessment of pulmonary sarcoidosis involves the measurement of pulmonary physiology [4], chest imaging techniques [4], and the use of biomarkers of disease activity and prognosis [5]. These assessments are labor-intensive, complex, and costly. Although eliciting symptoms of pulmonary sarcoidosis may lack the resolution of more objective assessments, it can be obtained easily and cheaply. Eliciting symptoms does not even require a scheduled visit with a clinician, as this can be performed in real time via electronic devices [6]. Most importantly, symptoms reflect quality-of-life issues of patients, which are a major treatment indication for sarcoidosis [7]. It is problematic to devise a treatment plan for a disease without taking into account how the patient feels. The ascertainment of pulmonary sarcoidosis symptoms can act as a gatekeeper for expensive and time-consuming sophisticated assessments to optimize their appropriate use and avoid their overuse.

As is the case for most diseases, the presence of pulmonary sarcoidosis symptoms is a more sensitive than specific marker of disease activity. The presence of pulmonary sarcoidosis symptoms may also have prognostic significance. This manuscript will review the major symptoms of pulmonary sarcoidosis and will describe their causes, treatment implications, and impacts on clinical outcomes.

## 2. Cough

Cough is an extremely common symptom of pulmonary sarcoidosis. In comparison to controls, an unselected group of pulmonary sarcoidosis patients was shown to have a markedly increased cough frequency and severity [8]. The reported frequency of cough varies widely across different sarcoidosis populations, and we suspect that this is, in part, related to the heterogeneity of the sarcoidosis cohorts that were analyzed in terms of disease activity, sarcoidosis treatment, and percentage of patients with significant pulmonary involvement. Given those caveats, the prevalence of cough in sarcoidosis patients has been estimated at between 3% and 53% [9]. Cough has been found to be more severe in black sarcoidosis patients than in white ones, and more severe and more prevalent in women with sarcoidosis than in men [9,10]. The severity of cough does not seem to be associated with spirometry, Scadding stage, smoking status, or age [8,10]. In one series of 36 consecutive exacerbations of pulmonary sarcoidosis (defined as worsening pulmonary symptoms, worsening spirometry, and no clinical evidence of an alternative cause of pulmonary worsening other than pulmonary sarcoidosis) [11], cough was present in 88 percent of the patients, and was more common than any other pulmonary symptom including dyspnea, wheeze, and chest pain [11]. Therefore, cough is a sensitive, although not a specific, finding of active pulmonary sarcoidosis, which we define as symptomatic disease caused by the granulomatous inflammation of sarcoidosis. It is problematic to determine if pulmonary sarcoidosis is active. Although chest scan imaging [12], cellular analysis of bronchoalveolar lavage [13], and pulmonary fludeoxyglucose F18 (FDG) uptake on positron emission tomography (PET) scanning [14] are fairly accurate in determining pulmonary sarcoidosis activity, they are expensive and/or invasive. Therefore, the clinical diagnosis of active pulmonary sarcoidosis is often based on less-specific clinical features such as the clinical presentation and presenting of symptoms. In this regard, the absence of cough significantly lowers, but does not eliminate, the possibility of active pulmonary sarcoidosis.

The characteristics of cough are variable in sarcoidosis depending depending on its etiology (vide infra). The cough may be non-productive or productive, and productive cough is more common in those with a high cough frequency [8]. Pulmonary sarcoidosis-related cough is significantly less frequent during sleep [8]. Cough is often chronic in sarcoidosis, with more than one half of pulmonary sarcoidosis patients experiencing a cough of greater than 8 weeks duration; additionally, a significant percentage experience cough for more than one year [8]. Therefore, an acute self-limiting cough syndrome that resolves without sarcoidosis therapy is unlikely to have been caused by active pulmonary sarcoidosis. Patients with pulmonary sarcoidosis who cough often identify environmental triggers including smoky environments, perfumes, and scents [8]. Other sensations related to cough include a tickle sensation or irritation in the throat [8]. 

Pulmonary sarcoidosis-associated cough is a common reason for patients to seek medical attention [15]. Using the Leicester Cough Questionnaire (LCQ), a patient-reported outcome quality-of-life measure of cough [16], sarcoidosis patients have been found to frequently experience a significant quality-of-life impairment related to cough [8,10,17]. In addition, a significant association has been found between cough-related quality-of-life impairment as measured using the LCQ and general quality-of-life impairment as measured using the short form-36 (SF-36) [17]. These data suggest that worsening cough significantly impacts quality of life in a large proportion of sarcoidosis patients. Quality of life is a major indication for the treatment of sarcoidosis [7]. However, physicians have tended to rely on objective measures such as forced vital capacity (FVC) or radiographic findings as clinical endpoints in pulmonary sarcoidosis [4,18], and the correlation between these endpoints and quality of life is poor [19]. As it has been demonstrated that FVC and radiographic findings do not correlate with the severity of cough in sarcoidosis [8,10] this suggests that the monitoring of cough may be an important clinical endpoint for pulmonary sarcoidosis. 

**Figure 1:**
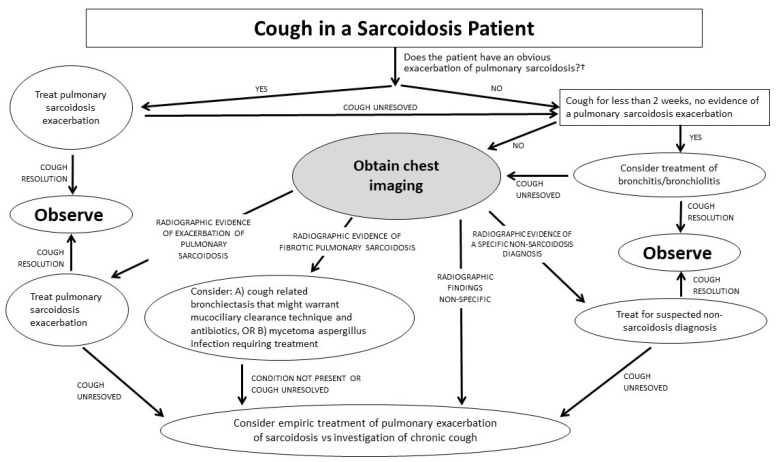
Proposed algorithm for the assessment of cough in a pulmonary sarcoidosis patient. †: Examples of such a scenario include (A) concomitant recurrence of sarcoidosis skin lesions or other extrapulmonary manifestations of sarcoidosis; (B) presentation very similar to the initial presentation of pulmonary sarcoidosis.

There are numerous potential causes of cough in pulmonary sarcoidosis. Probably the most common mechanisms causing pulmonary sarcoidosis-associated cough are airway irritation and mechanical airway damage caused directly by granulomatous inflammation. Sarcoid granulomas have a predilection for depositing in the airways [20] and the presence of endobronchial sarcoidosis lesions has been associated with cough [21,22]. This airway irritation/mechanical damage may cause an asthma-like syndrome [23] in which afferent nerve fibers are stimulated, thereby inducing cough [15,24]. Bronchial hyperreactivity with positive methacholine challenge testing is common in pulmonary sarcoidosis [25,26,27] and supports an asthma-like cough mechanism. However, sarcoidosis does not commonly cause an eosinophilic asthma condition, as serum IgE tends to be lower in sarcoidosis patients than in the general population [28] and levels of exhaled nitric oxide (eNO) are not increased with sarcoidosis [23]. It is also possible that the chronic cough of sarcoidosis is a primary disorder of sensory nerves, as is the case for other chronic cough syndromes [29]. “Cough reflex hypersensitivity” is the term used to describe this entity, and this has been demonstrated in pulmonary sarcoidosis patients using a capsaicin cough challenge test [8]. Airway distortion from fibrotic pulmonary sarcoidosis (Scadding Stage IV) may lead to significant bronchiectasis [30]. As with other forms of bronchiectasis, mucociliary clearance may be impaired, leading to cough which is often productive. Such patients are at increased risk of developing airway and parenchymal lung infection [31]. The acute onset or worsening of cough in these patients may signify an acute bronchitis, pneumonia, or an acute exacerbation of pulmonary sarcoidosis [23]. Sarcoidosis of the upper respiratory tract (SURT) may cause significant nasal sinus, laryngeal, or pharyngeal disease that may cause significant cough and other upper airway symptoms [32]. In addition, as previously mentioned, cough is not a specific symptom of pulmonary sarcoidosis and is a common complaint with a multitude of pulmonary disorders. The clinician should not assume that the development of cough in a pulmonary sarcoidosis patient is directly related to the disease and should include a search for an alternative explanation. A proposed algorithm for the assessment of cough in a pulmonary sarcoidosis patient is shown in Figure 1.

The measurement of cough is problematic because it is a multidimensional assessment involving both subjective and objective input. Cough frequency can be determined subjectively by the patient, but this method is not very accurate. Cough frequency monitors have been used in clinical trials [8,33]. Recently, smartphone-based artificial intelligence (AI) cough monitoring apps have become available that have the potential to accurately monitor cough frequency in clinical settings [34]. In addition, sound power and sound energy can now be measured as a non-invasive measure of cough intensity [34]. Although the frequency and intensity of cough may be important to measure in order to assess the effects of therapy, they do not accurately assess the impact of cough on the patient’s quality of life. Various health-related quality of life (HRQoL) patient-reported outcome measures (PROMs) of cough have been developed including the aforementioned Leicester Cough Questionnaire [16] (LCQ) and the Cough-specific Quality of Life Questionnaire (CQLQ) [35] both of which are well-validated and have had minimal clinically important differences (MCIDs) determined [33,35]. The LCQ has been used extensively in sarcoidosis [8,10,17]. Because these HRQoL cough PROMs consist of many items that require several minutes for the patient to complete, cough severity can also be assessed via a visual analog scale (VAS). VAS cough scales have been used in previous sarcoidosis trials [8,10] and they have been shown to correlate well with the LCQ in a large sarcoidosis cohort [10].

The treatment of pulmonary sarcoidosis-associated cough depends on its etiology. If cough is a symptom related to active pulmonary sarcoidosis, it usually responds to therapy for that condition, particularly with corticosteroid doses of 20 mg/day of prednisone or less [11]. Very frequently, cough and other pulmonary symptoms related to active pulmonary sarcoidosis respond in a matter of days. Because it is unlikely that sarcoid granulomas will appreciably resolve in this short period of time, it is likely that cough and many other symptoms of acute pulmonary sarcoidosis relate to the airway irritation or asthma-like mechanisms already described in this manuscript. Patients with fibrocystic sarcoidosis often develop cough from fibrosis-induced bronchiectatic airway changes [30] and this often requires bronchodilators and other mucociliary airway clearance techniques. These patients may frequently develop pulmonary infections [31] that require appropriate antimicrobial therapy to control cough. When cough is the most prominent symptom of pulmonary sarcoidosis and the patient does not appear to be experiencing a flare of the disease, inhaled corticosteroids (ICS) may be useful. The recommended doses in this situation are high: 800-1600 mcg/day of inhaled budesonide [36] or 800 mcg/day of inhaled fluticasone [37]. It is unclear if the benefits of ICS for pulmonary sarcoidosis-associated cough are related to a direct effect on granulomatous airway involvement or suppression of airway hyperreactivity. Although a meta-analysis suggests that ICSs are beneficial for pulmonary sarcoidosis-associated cough [38], a subsequent meta-analysis did not clearly show a benefit [39]. Although inhaled bronchodilators have not been extremely useful in chronic pulmonary sarcoidosis [40], they may augment suppression of cough in acute exacerbations of sarcoidosis if asthma-like mechanisms are present. Obviously, cough in pulmonary sarcoidosis patients may have an etiology unrelated to the disease that may require other therapies.

## 3. Wheezing

Wheezing is a very common symptom of pulmonary sarcoidosis. Wheezing was second only to cough as a symptom of acute pulmonary exacerbations of sarcoidosis [11]. Although pulmonary sarcoidosis is often classified as an interstitial lung disease that would be expected to result in restrictive lung physiology, airway obstruction is common in sarcoidosis and may occur via several mechanisms. The failure to appreciate mechanisms of airflow obstruction in pulmonary sarcoidosis frequently results in the disease being misdiagnosed as a highly prevalent obstructive lung disease such as asthma or chronic obstructive pulmonary disease [23].

**Figure 2:**
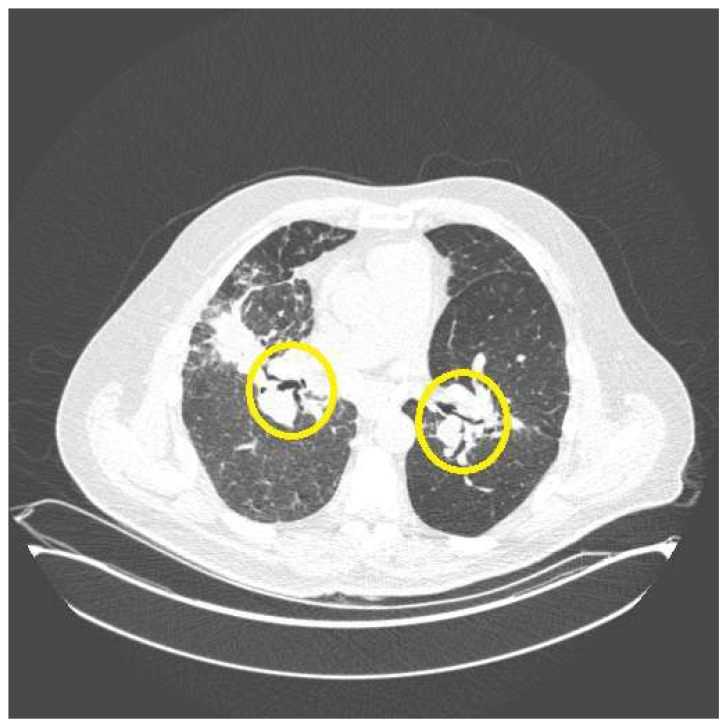
Chest CT scan of a patient with fibrotic pulmonary sarcoidosis. Distortion of airways (yellow circles) is common in this condition and is the result of granulomatous-induced airway fibrosis.

A major mechanism for airflow obstruction in sarcoidosis is from endobronchial sarcoid granulomas that may narrow, distort, or rarely completely obstruct the airway [41]. Airway involvement in sarcoidosis is common, as random endobronchial biopsies have demonstrated granulomatous inflammation in nearly 60 percent of pulmonary sarcoidosis patients [20]. As mentioned, granulomas may also irritate airways causing bronchospasm by stimulating afferent nerve fibers [8,15,29] or asthma-like mechanisms [23]. As mentioned, the fact that some patients with acute exacerbations of pulmonary sarcoidosis improve after a few days of therapy [42] suggests that bronchospasm, airway nerve fibers, or asthma-like mechanisms are involved, as this seems too rapid a response to attribute to the resolution of granulomatous inflammation. Endobronchial granulomas may result in significant airway scarring and distortion that often leads to airflow obstruction [12,30]. This is commonly seen in Scadding stage IV fibrocystic sarcoidosis (Figure 2). In fact, these fibrotic pulmonary sarcoidosis patients demonstrate significant airflow obstruction more frequently than all other forms [43]. The airflow obstruction in fibrotic pulmonary sarcoidosis occurs not only in the large airways, but also the small airways which contribute significantly to the pathophysiology [44]. Rare causes of airflow obstruction in pulmonary sarcoidosis include the development of significant bullous disease in fibrotic patients [45] and airway compression from mediastinal lymphadenopathy. Although the latter entity is common radiographically, it usually fails to cause significant airflow obstruction unless the lymph nodes are highly calcified [46]. Table 1 lists the common causes of airflow obstruction in pulmonary sarcoidosis.

Wheezing is problematic to quantify. Although smartphone-based artificial intelligence (AI) monitoring apps have been developed for cough [34], we are unaware that they have been developed to monitor wheezing. Obviously, airflow obstruction, the physiologic basis for wheezing, can be assessed using pulmonary function tests. Still, irrespective of the physiologic abnormalities associated with wheezing or the sound that is generated, wheezing does impact quality of life. We are unaware of specific HRQoL PROMs that address wheezing, although wheezing is a common item in general respiratory disease HRQoL PROMs [47]. We suspect that there is probably minimal benefit in specifically quantifying the severity of wheezing in pulmonary sarcoidosis, as it is problematic to measure and is usually associated with other clinical manifestations of the disease that are easier to monitor.

Wheezing is usually not specifically treated in pulmonary sarcoidosis. As wheezing is a manifestation of acute pulmonary exacerbations of sarcoidosis [11], systemic therapy for this condition (often oral corticosteroids initially) is usually effective in alleviating wheezing. Inhaled corticosteroids may be effective as has been described for pulmonary sarcoidosis-associated cough (vide supra). There are almost no clinical data on the use of beta agonist or anticholinergic inhalers in sarcoidosis. One study found that there was an improvement in forced expiratory volume in one second (FEV1) with salmeterol and ipratropium, with the former potentiated by concomitant budesonide inhalation [48]. Certainly, it would be prudent to consider adding a beta agonist inhaler to the standard pulmonary sarcoidosis treatment of a patient with prominent wheezing, although this maneuver has never been subjected to study. Wheezing from airflow obstruction related to endobronchial fibrosis and distortion in fibrotic pulmonary sarcoidosis would not be expected to respond to bronchodilators [49]. As such patients have significant bronchiectasis and retained airway secretions, they may benefit from mucociliary clearance techniques [30]. 

## 4. Dyspnea

Dyspnea is an extremely common symptom in pulmonary sarcoidosis patients. Dyspnea is more common in pulmonary sarcoidosis patients than in healthy matched controls, with moderate to severe dyspnea being more than 10 times more common (56% versus 4%) in the pulmonary sarcoidosis group [50]. Dyspnea was also the third most frequent symptom of acute exacerbations of pulmonary sarcoidosis behind cough and wheeze [11].

There are numerous causes of dyspnea in pulmonary sarcoidosis patients. These causes include the deposition of sarcoidosis granulomas in the lung, manifestations of fibrotic sarcoidosis, multiple mechanisms that can cause pulmonary hypertension, complications of treatment, manifestations of extrapulmonary sarcoidosis, psychosocial/functional issues, and conditions completely unrelated to sarcoidosis. These mechanisms of dyspnea are listed in Table 2. Obviously, these causes of dyspnea are so diverse that it mandates that the clinician rigorously evaluates the cause of dyspnea in a pulmonary sarcoidosis patient to ensure proper treatment for this symptom. In addition, the cause of dyspnea in a pulmonary sarcoidosis patient may be completely unrelated to the disease, and all of these causes should also be considered.

Dyspnea is a sensation that may affect or be affected by emotional, psychological, and social states. For this reason, these four conditions cannot be assessed in isolation, and changes in any one of them may affect all the others. The interdependence of these states has been specifically confirmed in sarcoidosis [51,52,53], and this implies that dyspnea is heavily influenced by non-physiologic factors in pulmonary sarcoidosis.

As dyspnea is a subjective measure, it is typically quantified via a PROM or VAS [54]. It has been clearly shown that the correlation between pulmonary function measurements and dyspnea is poor [19,51]. Therefore, pulmonary function test results cannot be reliably used as a surrogate for dyspnea. Specific dyspnea measures in sarcoidosis have included the Baseline Dyspnea Index (BDI) [51,55], Transitional Dyspnea Index (TDI) [51], the Borg Dyspnea Scale [51,56], and the Modified Medical Research Council (MRC) Dyspnea Scale [51]. The Modified MRC Dyspnea Scale has often been used as an entry criterion in clinical sarcoidosis trials [18]. Although these PROM and VAS measures of dyspnea function well in clinical trials of large cohorts of patients, most have significant variability that make them problematic to use longitudinally in individual patients to assess significant changes in dyspnea over time. Most clinicians do not use these dyspnea measures to make interventions in individual patients but rather to prompt a more thorough evaluation of the patient when dyspnea measures suggest a significant change. 

As there are innumerable causes of dyspnea, its treatment is dependent on its specific cause. Although exercise training regimens in sarcoidosis patients have been shown to improve the 6-min walk distance and lessen fatigue, they have no significant effect on dyspnea as measured using the Borg Dyspnea Scale [57]. Inspiratory muscle training in sarcoidosis patients has been demonstrated to significantly reduce dyspnea [58]

## 5. Chest Pain

Chest pain is a common symptom of pulmonary sarcoidosis. Chest pain has been reported as a presenting complaint in 9 percent of sarcoidosis patients [59], in 12 percent of patients experiencing an exacerbation of pulmonary sarcoidosis [11], and in 27 percent of patients with established sarcoidosis [60].

**Figure 3:**
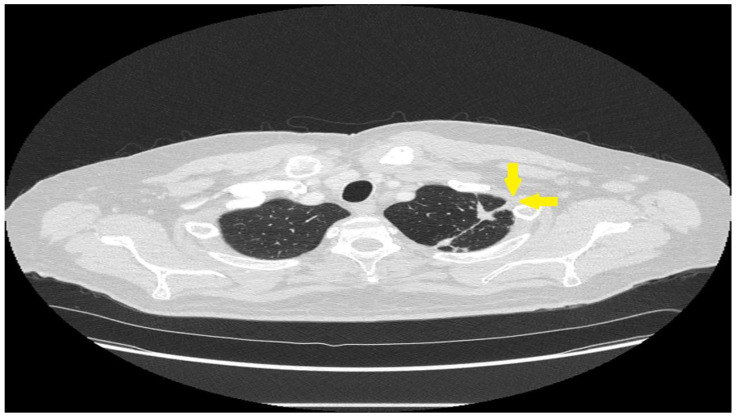
Although most chest pain in pulmonary sarcoidosis is unrelated to specific lung lesions, it may be related to pleural lesions on occasion. The CT scan shows an intraparenchymal pulmonary sarcoidosis lesion that extends to the pleura (arrows). This was the exact location of the patient’s pleuritic chest pain. The pain responded to anti-sarcoidosis therapy.

Pulmonary sarcoidosis-associated chest pain is usually pleuritic in character and is most common in the substernal and infrascapular areas [61]. The chest pain is often associated with coughing [61]. The pleuritic character of the pain and its association with cough suggests that it might be caused by cough-induced musculoskeletal irritation of the chest wall [61]. No correlation has been found between pulmonary sarcoidosis-associated chest pain and the following chest imaging features: Scadding chest radiograph stage, location of sarcoidosis-related lung nodules, mediastinal lymph node burden, and location of pleural disease [61]. However, although it is an anecdotal finding, we have identified specific pulmonary sarcoidosis patients with localized pleural disease that correlates well with the location of their pain (Figure 3). We therefore believe that pleural and subpleural sarcoidosis may lead to chest pain on rare occasions.

There are other causes of chest pain associated with sarcoidosis and pulmonary sarcoidosis. As mentioned, chest pain occurs in approximately 10 percent of patients with an exacerbation of sarcoidosis [11]. Patients with sarcoidosis-associated small fiber neuropathy may develop a sensation of numbness and burning in scattered locations, including the chest [62]. Sarcoidosis is associated with pulmonary embolism [63,64], which should be considered in sarcoidosis with the sudden onset of dyspnea and pleuritic chest pain. Rarely, a pneumothorax can occur with pulmonary sarcoidosis from necrosis of subpleural granulomas or rupture of a cystic lesion in a patient with Scadding stage 4 fibrocystic disease [65]. Sarcoidosis-associated pleural effusion is a rare event but may cause chest pain [66]. 

The treatment of pulmonary sarcoidosis-associated chest pain depends on its etiology. If chest pain develops with an acute pulmonary exacerbation of sarcoidosis, it usually responds to therapy for this condition [11]. The aforementioned common presentation of pulmonary sarcoidosis-associated chest pain with pleuritic substernal or infrascapular pain often responds to non-steroidal anti-inflammatory agents.

There are innumerable alternative causes of chest pain in pulmonary sarcoidosis patients that are unrelated to the disease. The clinician should diligently explore these possibilities and not reflexively assume that the chest pain in pulmonary sarcoidosis is caused by sarcoidosis.

## 6. Hemoptysis

Hemoptysis is a rare symptom of pulmonary sarcoidosis. The most comprehensive review of hemoptysis in pulmonary sarcoidosis was published more than 35 years ago, and found that six percent of 433 sarcoidosis patients developed hemoptysis over the course of their disease [67]. A literature review of 144 cases of sarcoidosis-associated hemoptysis found that the reported incidence of hemoptysis in sarcoidosis varied from 1 to 11 percent [68]. Only 22 percent (31/144) of these patients had a bronchoscopy examination reported, which was normal in one-third of them and in the remainder revealed mucosal thickening, hyperemia, congestion, and/or narrowing of the airways [68]. Interestingly, most of these patients had Scadding stage 1 (bilateral hilar adenopathy without parenchymal opacities) or stage 2 (bilateral hilar adenopathy and parenchymal opacities without fibrosis) chest radiographs. Hemoptysis resolved in most of these cases, with only one death and five recurrences reported.

Hemoptysis may rarely be an initial manifestation of sarcoidosis, with only four percent of 433 patients presenting with this symptom [67]. Scattered reports of hemoptysis at the onset of sarcoidosis suggest that it usually resolves with a course of corticosteroids [68,69,70]. A biopsy from an endobronchial lesion in one such case revealed “non-caseating granulomas with central necrosis” [68], and we suspect that airway necrosis related to granulomatous inflammation is the most common cause for this presentation. This mechanism is consistent with the fact that hemoptysis at presentation of sarcoidosis usually responds to corticosteroids.

**Figure 4:**
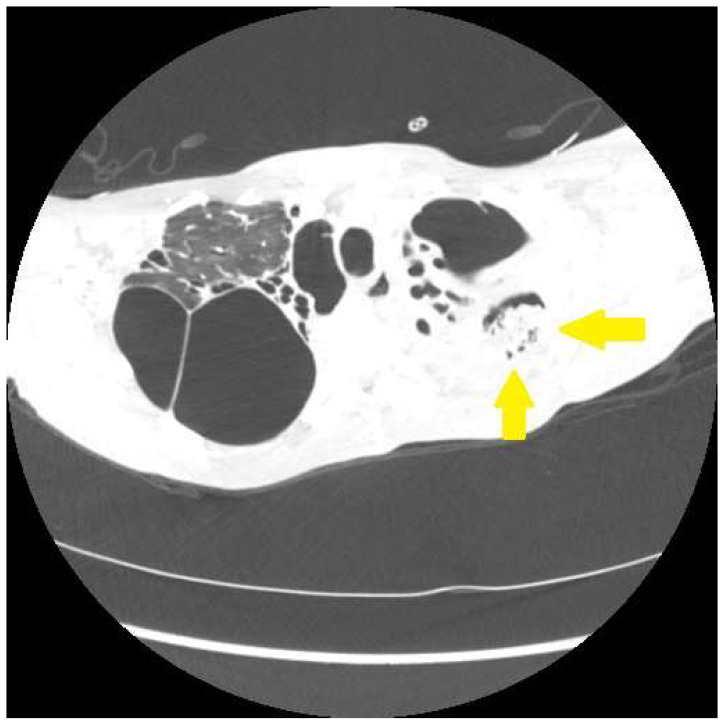
Chest CT scan of a fibrocystic pulmonary sarcoidosis patient demonstrating a mycetoma (arrows). These are often associated with significant pleural thickening.

Hemoptysis may occur in patients with Scadding stage 4 fibrocystic sarcoidosis via a number of mechanisms. Sarcoidosis-associated bronchiectasis occurs in up to 50 percent of patients with fibrotic sarcoidosis [30,31]. This bronchiectasis is usually of the traction type, and is most prominent in the central airways (Figure 2) [30,71]. Bronchiectasis in fibrotic sarcoidosis is most likely related to airway fibrosis caused by granulomatous inflammation of the airways [72]. Bronchiectasis may lead to airway infection causing significant hemoptysis [73]. Chronic aspergillus infection including aspergilloma, chronic cavitary pulmonary aspergillosis, and chronic fibrosing aspergillosis commonly occurs with pulmonary sarcoidosis, and almost exclusively in those with fibrocystic disease [30,74]. These patients frequently present with hemoptysis that may be life-threatening (Figure 4) [75]. Although pulmonary hypertension may be seen with any radiographic presentation of sarcoidosis [76], it is most common in those with fibrocystic disease [76]. Pulmonary hypertension may result in significant hemoptysis by causing vascular engorgement and a hemorrhagic diathesis [77]. The management of hemoptysis in fibrocystic sarcoidosis involves rapidly identifying the cause and quickly administering treatment. We have a low threshold for initiating antibiotics empirically for a bronchiectasis-related infection. We also routinely obtain respiratory samples to evaluate for infection. Imaging or microbiologic evidence of aspergillus infection should prompt the obtaining of pulmonary specimens and serologies for the identification of these pathogens. If the fungal disease is localized, bronchial artery embolization is a temporizing procedure that may acutely control the bleeding [78,79], although antifungal agents [74], transcutaneous instillation of antifungals [75], or surgical resection [74] may be required for long-term control. These patients should be evaluated for pulmonary hypertension, as this can not only directly cause hemoptysis [80] but may also exacerbate hemoptysis in patients who have an alternative primary cause of this symptom [75]. Obviously, fibrocystic sarcoidosis patients may develop hemoptysis from numerous other causes that are not associated with this specific form of the disease; these should be searched for and treated.

Other causes of hemoptysis in sarcoidosis include necrotizing sarcoid granulomatosis (NSG), a condition where the granulomas are typically confluent and necrotic [68,81]. The necrosis may be the result of granulomas that deposit around pulmonary vessels that are compressed leading to parenchymal lung infarction and necrosis [82]. Radiographically, NSG often demonstrates multiple pulmonary nodules with cavitation [83]. It is currently unclear as to whether NSG is a specific disease entity or a form of sarcoidosis [82]. The rare entity of sarcoidosis-related pulmonary veno-occlusive disease has also been reported to present with recurrent hemoptysis [84].

Several medical conditions are associated with sarcoidosis that may lead to hemoptysis. Sarcoidosis patients appear to be at a higher risk of pulmonary embolism [63,85,86] and lung cancer [87,88], both of which often present with hemoptysis. Immunosuppressive agents used to treat sarcoidosis may increase the risk of necrotic lung infection that may cause hemoptysis.

Table 3 lists several causes of hemoptysis that are directly or indirectly related to pulmonary sarcoidosis. The clinician should be aware that causes of hemoptysis not related to sarcoidosis may also occur in these patients.

## 7. Pulmonary Sarcoidosis without Pulmonary Symptoms

It has been estimated that 50 [59,89] to 85 [90] percent of pulmonary sarcoidosis patients present without pulmonary symptoms. Some of these patients are diagnosed with pulmonary sarcoidosis fortuitously via chest imaging studies performed for other reasons. Although approximately 50 percent of pulmonary sarcoidosis cases can be discovered in this way via mass population chest radiograph screenings [91], this occurs in less than 10 percent of pulmonary sarcoidosis patients cared for in clinical practices [89,90,92]. Approximately one-quarter of pulmonary sarcoidosis patients present with isolated symptoms of extrapulmonary organ involvement (e.g., eye symptoms, skin lesions) [89]. Lofgren’s syndrome, consisting of bilateral hilar adenopathy on a chest radiograph, erythema nodosum skin lesions, and commonly fever and an ankle periarthritis, [93,94] is a common presentation of sarcoidosis and pulmonary symptoms are often absent. The frequency of Lofgren’s syndrome is quite variable throughout the world, being particularly common in Northern Europe and rare in Spain and Japan [95]. In up to 50 percent of cases, pulmonary sarcoidosis may present with constitutional symptoms such as fever, malaise, night sweats, and weight loss that are not attributable to a specific organ [59].

Patients with asymptomatic pulmonary sarcoidosis most commonly have no evidence of parenchymal lung disease on a chest radiograph, with either a normal chest radiograph (Scadding stage 0) or bilateral hilar/mediastinal lymphadenopathy without parenchymal opacities (Scadding stage 1) [96,97]. The spirometry of asymptomatic pulmonary sarcoidosis patients is normal in more than 90 percent of cases [96,97].

The prognosis of asymptomatic pulmonary sarcoidosis is better than that of symptomatic patients. In one series of 660 sarcoidosis patients where 175 (27%) were asymptomatic and at least 145 (83%) of those had pulmonary sarcoidosis, asymptomatic patients less frequently required treatment, developed less organ involvement, and had improved health-related quality of life [98].

## 8. Summary

Although the symptoms associated with pulmonary sarcoidosis are not specific for the disease, they provide important clinical insights. Cough is the most frequent symptom of active pulmonary sarcoidosis, and the lack of cough greatly lowers the probability of an exacerbation of pulmonary sarcoidosis. Wheezing is underappreciated in pulmonary sarcoidosis. It is a very common symptom in acute pulmonary sarcoidosis from the granulomatous involvement of the airways and in chronic sarcoidosis from airway distortion from fibrosis. Chest pain is also an underappreciated symptom of pulmonary sarcoidosis. It is typically pleuritic and usually not associated with the severity of the disease. Hemoptysis is a relatively uncommon initial symptom of pulmonary sarcoidosis that usually responds well to corticosteroids. However, hemoptysis in chronic fibrotic pulmonary sarcoidosis may suggest bronchiectasis, aspergillus infection, or pulmonary hypertension; all of which are serious and potentially life-threatening disease complications. Asymptomatic pulmonary sarcoidosis patients tend to have minimal disease on chest imaging, less often require treatment, and have a better long-term quality of life than symptomatic patients.

The assessment of the symptoms of pulmonary sarcoidosis is easy to obtain and has no cost. We believe that comprehensive knowledge of these symptoms will aid the clinician in identifying and managing various manifestations of the disease. Advances in artificial intelligence may allow for more accurate monitoring of these symptoms that may significantly improve patient management.

## Figures and Tables

**Table 1:** Common causes of airflow obstruction in sarcoidosis (that could induce wheezing).

Cause	Form of Pulmonary Sarcoidosis
Endobronchial deposition of granulomas	Active pulmonary sarcoidosis
Cough reflex hypersensitivity granulomatous inflammation of airways	Active pulmonary sarcoidosis
Bronchospasm from granulomatous inflammation of airways	Active pulmonary sarcoidosis
Airway fibrosis from previous granulomatous inflammation of airways	Fibrotic pulmonary sarcoidosis
Development of bullous disease/emphysema *	Fibrotic pulmonary sarcoidosis
Airway compression from mediastinal lymphadenopathy *^,^†	mediastinal lymphadenopathy from sarcoidosis

* Rare; † This manifestation most commonly occurs in patients with significantly calcified mediastinal lymphadenopathy.

**Table 2:** Common causes of dyspnea in sarcoidosis.

Major Category	Mechanism	Mechanism of Dyspnea	Treatment
**Intrathoracic deposition of granulomas**	Intrapulmonary deposition of granulomas in alveoli/ interstitium	Decreased lung compliance	Anti-granulomatous therapy
	Intrapulmonary deposition of granulomas in airways	Increased airway resistance	Anti-granulomatous therapy
	Asthma/bronchospasm	Increased airway resistance	Anti-granulomatous therapy; anti-asthma therapy
	Sarcoidosis-associated pleural effusion (deposition of granulomas in the pleura) *	Decreased lung volume, overdistention of respiratory muscles	Anti-granulomatous therapy
**Pulmonary fibrosis**	Fibrosis in alveoli/ interstitium	Decreased lung compliance	Anti-fibrotic therapy?; Anti-granulomatous therapy?
	Fibrosis in airways	Increased airway resistance	Anti-fibrotic therapy?; Anti-granulomatous therapy?
**Multiple processes: Intrapulmonary deposition of granulomas, pulmonary fibrosis, hypoxic pulmonary vasoconstriction from parenchymal sarcoidosis**	Pulmonary hypertension	Increased pulmonary vascular resistance, hypoxemia	Pulmonary vasodilators; anti-granulomatous therapy?
**Complications of treatment**	Weight gain from corticosteroids	Restrictive ventilatory defect	Weight loss; reduce corticosteroid dose if possible
	Pulmonary infection	Immunosuppressive medications	Treat the infectious pathogen; reduce immunosuppression if possible
	Respiratory muscle weakness	Corticosteroid myopathy	Reduce corticosteroid dose
	Ischemic/hypertensive cardiomyopathy	Corticosteroid-induced hypertension/diabetes	Treatment of hypertension/diabetes and ischemic heart disease; reduce corticosteroid dose if possible
**Extrapulmonary sarcoidosis**	Cardiac sarcoidosis	Cardiomyopathy	Anti-granulomatous therapy for cardiac sarcoidosis
	Respiratory muscle involvement with sarcoidosis *	Respiratory muscle failure	Anti-granulomatous therapy for respiratory muscle sarcoidosis
	Pulmonary embolism	Hypoxemia, increased pulmonary vascular resistance	Anti-granulomatous therapy for respiratory muscle sarcoidosis
**Psychological/emotional/physical state associated with sarcoidosis**	Multiple mechanisms: depression, fatigue, cognitive impairment	Increase in the sensation of dyspnea	Treat the underlying mechanism
**Process unrelated to sarcoidosis**	Innumerable mechanisms	Innumerable etiologies	Treat the underlying mechanism

?: questionable/controversial treatment; *: rare.

**Table 3:** Causes of hemoptysis in pulmonary sarcoidosis.

Cause	Form of Sarcoidosis	Mechanism
Granulomatous airway lesions	Active pulmonary sarcoidosis (granulomatous inflammation)	Granulomatous necrosis of an airway lesion
Bronchiectasis	Fibrocystic sarcoidosis	Bronchiectasis from airway fibrosis from previous granulomatous inflammation. Hemoptysis from infectious bronchitis/bronchiectasis
Aspergilloma/Chronic aspergillus lung infection	Fibrocystic sarcoidosis	Aspergillus colonization of devitalized lung with subsequent locally invasive disease
Pulmonary hypertension	Many forms of sarcoidosis, most commonly fibrocystic disease	Pulmonary hypertension leads to vascular engorgement and a hemorrhagic diathesis that may be exacerbated by infection, granulomatous inflammation
Necrotizing sarcoid granulomatosis	Necrotizing sarcoid granulomatosis—unclear if this is a form of sarcoidosis or a separate disease entity	Parenchymal necrosis
Pulmonary embolism	Associated with sarcoidosis epidemiologically	Pulmonary infarction; pulmonary hypertension
Lung cancer	Associated with sarcoidosis epidemiologically	Parenchymal/Airway necrosis
Pulmonary infection	Associated with immunosuppressive agents used to treat sarcoidosis	Parenchymal/Airway necrosis
Hemoptysis not specifically related to sarcoidosis	Not applicable	Not applicable
